# The X-like shaped spatiotemporal structure of the biphoton entangled state in a cold two-level atomic ensemble

**DOI:** 10.1038/srep42373

**Published:** 2017-02-20

**Authors:** Dasen Zhang, Zhiming Zhang

**Affiliations:** 1 Guangdong Provincial Key Laboratory of Nanophotonic Functional Materials & Devices (SIPSE), and Guangdong Provincial Key Laboratory of Quantum Engineering & Quantum Materials, South China Normal University, Guangzhou 510006, China

## Abstract

We study the spatiotemporal structure of the biphoton entangled state generated by the four-wave mixing (FWM) process in a cold two-level atomic ensemble. We analyze, for the first time, the X-like shaped structure of the biphoton entangled state and the geometry of the biphoton correlation for different lengths and densities of the cold atomic ensemble. The propagation equations of the photon pairs generated from FWM process are derived in a spatiotemporal framework. By means of the input-output relations of the propagation equations, the biphoton amplitude function is obtained in a spatiotemporal domain. In the given frequency range, the biphoton amplitude displays an X-like shaped geometry, nonfactorizable in the space-time domain. Such an X-like shaped spatiotemporal structure is caused by the phase matching and the FWM gain. The former leads to the X-like shaped envelope of the biphoton correlation, while the latter gives rise to the oscillations around the X-like shaped envelope.

Entangled paired photons are not only of interest on interference phenomenon but also have many potential applications, such as quantum computing and quantum communication^1^, quantum imaging^2^, quantum lithography^3^ and quantum measurement^4^. Traditionally, entangled photon pairs are produced from spontaneous parametric down-conversion (SPDC) in a noncentrosymmetric crystal^5^. In the past decade, paired photons generated from four-wave mixing (FWM) process in cold atoms attract a great deal of attention^6^, because they have a longer coherence time (compared with that in SPDC process). Wen *et al*. have explored the transverse effects of the entangled paired-photon in refs 7 and 8. However, a few studies have been reported on two-photon coherence in both spatial and temporal domain (i.e. spatiotemporal domain).

In recent years, Gatti *et al*. have found an X-shaped spatiotemporal structure of biphoton correlation, generated by the SPDC process in BBO crystal^9^. Jedrkiewicz *et al*. have proven that the X-shaped spatiotemporal coherence is to arise in multidimensional optical system involving nonlinear wave propagation in normal dispersion^10^,^11^. Inspired by their seminal work, we study the space-time coherence of biphoton generated in a cold atomic medium by FWM. In the Heisenberg picture, we deduce the propagation equations of paired photons in the idealized two-level atomic system and obtain the biphoton amplitude function in spatiotemporal domain. This gives a theoretical model to deal with the biphoton coherence in space-time domain. It turns out that the biphoton amplitude also displays the similar X-shaped spatiotemporal coherence structure in a limited frequency range. This result is very interesting but not difficult to understand, since the optical system we choose has some properties like multi-level normal dispersion medium in the given frequency range^10^,^11^. To distinguish the spatiotemporal structure of the biphoton amplitude in the atomic ensemble with FWM process from the X-shaped structure in the crystal with SPDC process, we name the former the X-like shaped structure. However, when it does not cause confusion, for simplicity in terminology, we call both of them the X structure (or X correlation). To our knowledge, this is the first report on studying the X structure of the biphoton correlation in atomic medium.

As shown in Fig. 1, while two copropagating pump waves act on a cold atomic ensemble^7^, the two conjugated optical fields (i.e. the Stokes and anti-Stokes beams) are spontaneously produced, and propagate in the same direction. Under the conditions of the energy conservation and the momentum conservation, the biphoton amplitude shows an X-like shaped geometry structure, which is analogous to the X-shaped biphoton amplitude structure in SPDC^9^. It is important to emphasize that such an interesting X-like shaped correlation is nonseparable in space-time domain. So we need to study this X-like shaped structure of biphoton amplitude in the complete spatiotemporal domain.

To get insight into the nonseparable X correlation, on the one hand, we compare the X structure in the atomic medium (by FWM) with the one in BBO crystal (by SPDC); on the other hand, we analyze the evolution of X structure with different lengths and densities of the atomic medium. Eventually, we realize that the X correlation is under control of the FWM gain and the phase-matching relation.

## Methods

We assume that the pump beam is classical, and denote it as


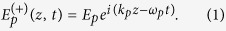


The Stokes and anti-Stokes fields are taken as quantized^12^, respectively,


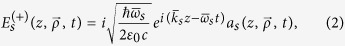



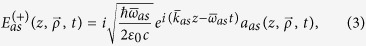


where *z* and 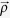 denote the longitudinal and the transverse components of the coordinate vector, respectively. 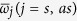 are the central frequencies of Stokes and anti-Stokes photons, and 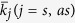 are their central wave numbers in the medium. *ε*_0_ and *c* are respectively dielectric constant and velocity of light in the vacuum. 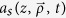 and 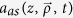 are the annihilation operators of the Stokes and anti-Stokes fields and are given by^12^


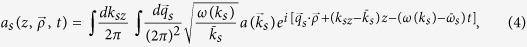



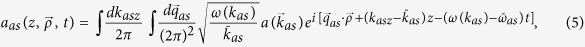



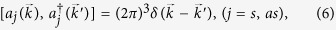


here *k*_*jz*_ and 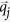 (*j* = *s, as*) are the longitudinal and transverse components of the wave vector, 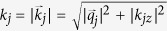. In a finite spatial region of volume V, the free Hamiltonian and interaction Hamiltonian for the FWM process take the form^6^,^12^


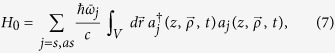



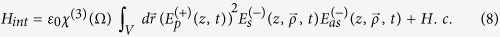


For simplicity, we consider that the paired photons are created in a cold atomic ensemble, in which all the dephasing rates in atomic ensemble are much less than the pump detuning Δ_*p*_. Under this condition, the nonlinear and linear susceptibility for the generated Stokes and anti-Stokes fields can be real^7^,^13^:


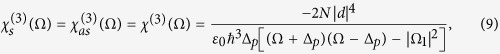



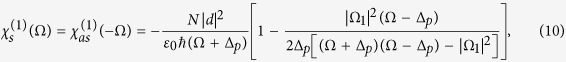


where 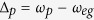, 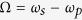, 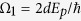. *N* characterizes the density of the cold atom, *d* is the dipole matrix element. Since the Stokes and anti-Stokes beams are mutual conjugate waves, we only need to calculate the evolution of the Stokes or anti-Stokes wave, and the other can be obtained by the same way. The evolution of the slowly varying amplitudes 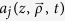 is described by the following equation^14^:


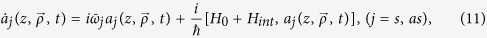


where the first term is from the time dependence 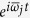 included in the definitions (4) and (5) of operator 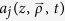
12,15.

By expanding Eq. (11), and then after some long calculations (see Supplementary Information), we obtain the propagation equations of the Stokes and anti-Stokes beams


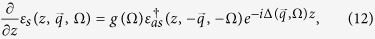



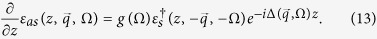


The above equations are the coupled wave equations of the Stokes and anti-Stokes wave without the linear absorption, which have the same form as the ones in ref. 13 without absorption.

Next, by using the input-output solutions of Eqs (12) and (13) (see Supplementary Information), we can compute the biphoton amplitude^16^


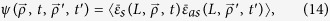


where 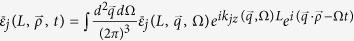, (*j* = *s, as*), and *L* is the length of the atomic ensemble. Then we can obtain


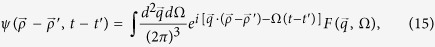


where


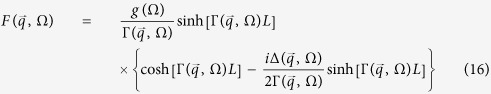


and


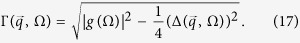


In the above equation, we adopt the phase-mismatch relationship^17^


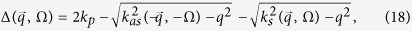


where 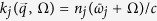, 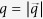, and 
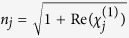
, (*j* = *s, as*). By means of a Fourier-Hankel transform, equation (15) can be rewritten as ref. 16:


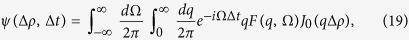


where 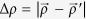, Δ*t* = *t* − *t*′, and *J*_0_ (*q*Δ*ρ*) is the zero-order Bessel function of the first kind, which can be written as 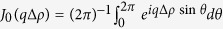.

In the following sections we will use equation (19) to study the spatiotemporal structure of the biphoton amplitude.

## Results

In order to obtain quantitative results, we analyze the outcomes of the biphoton amplitude’s structure by evaluating *ψ* numerically and the parameters used here are *λ*_*p*_ = 780 *nm*, Δ_*p*_ = −2*π* × 83 *MHz, d* = 3.6 × 10^−29^ *C*·*m*, Ω_1_ = 2 *MHz, N* = 2 × 10^17^ *atoms/m*^3^ 7. Because all the lasers used are near the atomic resonance here^18^ (on the ^87^*Rb* D2 line), for convenience, we make 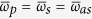. As predicted, by selecting an appropriate frequency range from 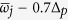 to 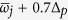 (*j* = *s, as*), we can get the X-like shaped structure of the two-photon amplitude, which is similar to the one in the SPDC process, and then we investigate how the X structure of the biphoton amplitude changes with the length and the density of the atomic medium.

### X-like shaped biphoton amplitude in a cold atomic medium

Figure 2(a,b) show the two-dimensional and three-dimensional structures of the biphoton amplitude |*ψ*(Δ*ρ*, Δ*t*)|, respectively. As shown in Fig. 2(a), the biphoton amplitude displays its X-like shaped geometry apparently, which is inseparable in the space-time domain. There are many secondary peaks around the X-like shaped envelope. Especially, some small peaks arise along the temporal coordinate at 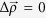, which is quite different from the spatiotemporal coherence picture in the SPDC. In fact, the whole X-like shaped structure consists of two components. One is the X-shaped envelope, and the other is the oscillations around the X-shaped envelope. The physics of the X-shaped correlation can be understood as follows. While we detect the twin photons from the same position, namely 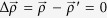, we are most likely to find them together in few nanoseconds. In contrast, while we collect the twin photons from different positions 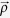 and 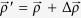, we are most likely to capture them by a certain delayed time 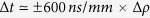. The dashed lines in Fig. 2(a) are given by the equations 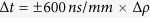, which are suitable to the legs of the X structure.

In fact, the X envelope is determined by the forward phase-matching condition in Eq. (18). As derived by Gatti *et al*.^9^,^19^, from Eq. (18) we can obtain the relation^19^


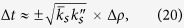


where 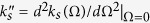. It can be seen that the temporal separation of twin photons increases with increasing their spatial separation and they are correlated. Since the twin photons have different frequencies 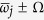 (*j* = *as, s*), they have different group velocities, which leads to their temporal separation in the output plane. Since they have different transverse wave vectors ±*q* (caused by the momentum conservation), their spatial separation is determined by diffraction. Meanwhile, their different frequencies and transverse wave vectors, which are caused by the phase-matching, lead to their different group velocities and diffractions, respectively.

**Figure 1:**
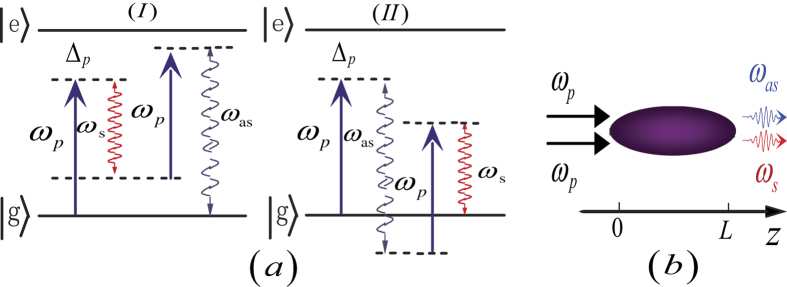
(**a**) Two types of four-wave mixing processes in a two-level atomic system. In the presence of two forward pump beams at the same frequency *ω*_*p*_, the two-level atoms generate two conjugate optical fields at the frequencies *ω*_*s*_ = *ω*_*p*_ + Ω and *ω*_*as*_ = *ω*_*p*_ − Ω. The pump detuning is Δ_*p*_ = *ω*_*p*_ − *ω*_*eg*_. (**b**) The twin photons created in the atomic ensemble with a length of L propagate in the same direction. The planes of *z* = 0 and *z* = *L* are the input plane and the output plane of the atomic medium, respectively.

**Figure 2:**
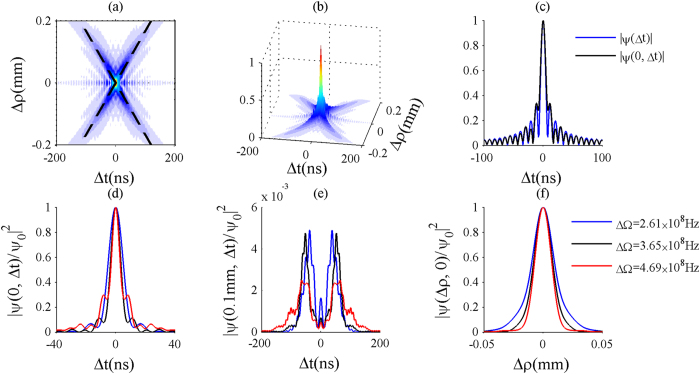
(**a**) Two-dimensional and (**b**) three-dimensional plots of 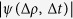, normalized to its peak value. (**c**) 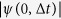 (black) and 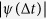 (blue). (**d**) Cut of the probability distribution along the temporal axis, showing the temporal correlation of photon pairs collected from the same position Δ*ρ* = 0 for different bandwidths ΔΩ of the frequency filter. (**e**) Same plot as in (**d**) for Δ*ρ* = 0.1 *mm*. (**f**) Cut of the probability distribution along the spatial axis, for different bandwidths of the frequency filter. In the case of a 10-mm-long cold *Rb* atomic-gas medium, and pumped at *λ*_*p*_ = 780 *nm*.

In contrast to the SPDC model^16^, the main difference is that the biphoton correlation exhibits the oscillation^20^, when the spatial separation is near zero (namely, 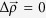). In Fig. 2(c), we plot the absolute values of the biphoton amplitudes *ψ*(0, Δ*t*) and 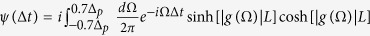. According to momentum conservation, only when *q* = 0, i.e., in the case of collinear propagation, photon pairs would exit form the atomic medium without spatial separation (Δ*ρ* = 0). Therefore *ψ*(0, Δ*t*) is indeed the biphoton amplitude in the case of collinear propagation. The *ψ*(Δ*t*) is the biphoton amplitude *ψ*(Δ*ρ*, Δ*t*) in the limiting case of perfect phase matching 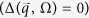, so only the FWM gain is in action. As shown in Fig. 1(a), there is an interference between the two FWM processes in a two-level atomic system^7^,^20^. In consequence, we can see that the *ψ*(Δ*t*) shows the oscillations, even if the effect of the phase mismatching on the biphoton amplitude is ignored. In Fig. 2(c) we compare the two curves that match very well. So we can conclude that the oscillation along the temporal axis is mainly caused by the interference between the FWM processes. In comparison, the diagram of the biphoton amplitude in the SPDC has no such an oscillation, since the SPDC gain is a constant and no interference appear in the SPDC process. Besides, there are some tiny oscillations around the wings of the X envelope (as shown in Fig. 2(a,b)), which is also caused by the interference effect of the two FWM processes. This is another difference compared with the SPDC model^16^, as shall be discussed in the next two sections.

Furthermore, in Fig. 2(d,e) we plot cuts of the probability distribution |*ψ*(Δ*ρ*, Δ*t*)|^2^ along the temporal axis for Δ*ρ* = 0 and Δ*ρ* = 0.1 *mm* for different widths of the frequency filter. When twin photons are collected at the same position, there are a series of oscillations in Fig. 2(d). In contrast, for Δ*ρ* = 0.1 *mm*, two symmetric temporal peaks appear in Fig. 2(e), surrounded by many small oscillations, which come from the interference between the two FWM processes. In both cases, the uncertainty on the arrival time of the second photon with respect to the first one is on the order of tens of nanoseconds, which is different from the SPDC model whose correlation time is on the order of few femtoseconds. However, similar to the SPDC, the correlation time is inversely proportional to the bandwidth of the frequency filter used. It is important to stress that in Fig. 2(e) two symmetric peaks mainly come from the phase matching, whereas the small oscillations are due to the interference effect of the FWM processes. In Fig. 2(f) we show the spatial profiles of the probability distribution |*ψ*(Δ*ρ*, Δ*t*)|^2^ for different bandwidths of the frequency filter, when the relative arrival time of photon pairs is resolved at Δ*t* = 0. In that case, we observe a correlation length nearly about 50 um that also scales inversely with the bandwidth of the frequency filter. As has been pointed out before, the biphoton amplitude curves in space and time are not only due to the phase-matching mechanism and the FWM gain, but also modulated by the frequency filter.

Before moving to the next section, let us make a short summary. The whole X-shaped correlation diagram is determined by the phase-matching condition and the FWM gain. The phase-matching leads to an X envelope of the biphoton correlation, whereas the interference between the FWM processes gives rise to the oscillations around the X envelope of the biphoton correlation. After revealing the X-shaped spatiotemporal structure of the biphoton amplitude, we study how it varies for different lengths and densities of the atomic medium.

### Biphoton correlation for different lengths of the atomic medium

In the previous section, we described the X-shaped spatiotemporal structure of the biphoton correlation in a cold atomic ensemble and discussed the probability distribution 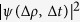 at the fixed relative spatial position. However, we are very interested in the integrated probability distribution 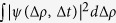 when the photons are collected from the whole cross section, without discriminating their positions. Next, we shall analyze how the biphoton amplitude 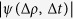, the probability distibution 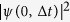 in the case of Δ*ρ* = 0, and the integrated probability distribution 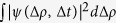 vary with the length of the atomic medium.

**Figure 3:**
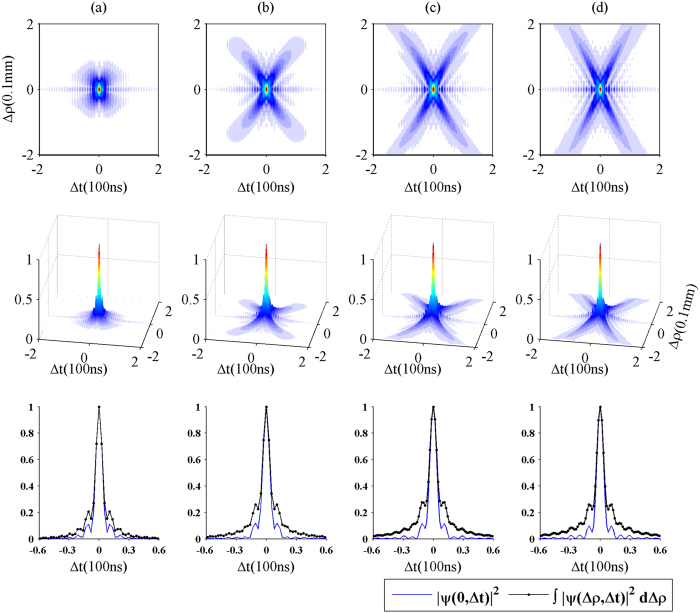
Plot of 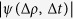 for different lengths of the atomic medium: (**a**) 2 mm, (**b**) 5 mm, (**c**)10 mm, and (**d**) 20 mm. The first row reports the 2D structures of the biphoton amplitudes, and the second row presents the 3D structures of the biphoton amplitudes. Other parameters are the same as in Fig. 2. The last row compares the probability distribution when the paired photons are collected from the same position (blue solid line), with the integrated probability distribution obtained without discriminating their positions (black dotted line).

Figure 3 shows the effects of the length of the atomic medium on the biphoton amplitude. The upper panels plot the two-dimensional structures of the biphoton amplitudes with different lengths of the atomic medium, and the central panels present their corresponding three-dimensional structures. From (16) and (19), we can see that 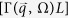 is an important quantity to the biphoton amplitude. It can be rewritten as 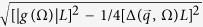. So, increasing the length of the medium, the phase mismatching and the FWM gain make more contribution to the X-shaped correlation. As shown in Fig. 3, the legs of X envelope become clearer and clearer with increasing the length of the atomic ensemble. This can be understood as follows. The temporal separation and spatial separation of the photon pairs come from their different group velocities and diffractions, respectively. When the atomic medium is short the photon pairs have very little time to separate in space. As a consequence, we can hardly observe an obvious X envelope in Fig. 3(a). However, when the medium becomes longer, twin photons take more time to reach the output plane of the medium and have enough time to separate in space and time. In this case, more and more twin photons are created and then populate not only the wings of the X envelope, but also the secondary oscillations around the X structure (see Fig. 3). These oscillations are caused by the interference between the FWM processes. Since the FWM gain *g*(Ω) we chose is not large, the oscillations increase slowly with increasing the length of the medium. From the above, the X envelope determined by phase matching extends substantially, while the oscillations caused by the interference effect of the FWM processes increase slightly. Therefore, with larger length of the atomic medium, the biphoton correlation is mainly controlled by the phase matching.

The lower panels of Fig. 3 present the comparison between the probability distribution 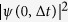 (the photons are taken from the same position) and the integrated probability distribution 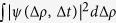 (the photons are collected without discriminating their positions). When the atomic medium is thin (*L* = 2 *mm*), the curves in Fig. 3(a) for the probability distribution and the integrated probability distribution are similar and the gap between them is not large, since there is not enough time for the twin photons to isolate in space, as mentioned above. Moreover, we calculate the standard deviation *σ* of the 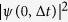 and the 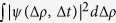, as shown in Table 1^16^. It can be seen that as the length of the atomic medium changes from 2 mm to 20 mm, the standard deviation of the integrated probability distribution increases from 21.7 ns to 35.9 ns, while that of the probability distribution for Δ*ρ* = 0 does not change so much. This is because when the FWM gain *g*(Ω) is small, the effect of *g*(Ω)*L* to the biphoton amplitude is also small. In the next section, we will find that the atomic density has a more noticeable influence on the probability distribution 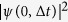.

### Biphoton correlation for different densities of the atomic medium

In this section, we explore how the structure of the biphoton amplitude 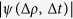, the probability distribution 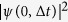 and the integrated probability distribution 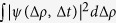 change with the atomic density.

**Figure 4:**
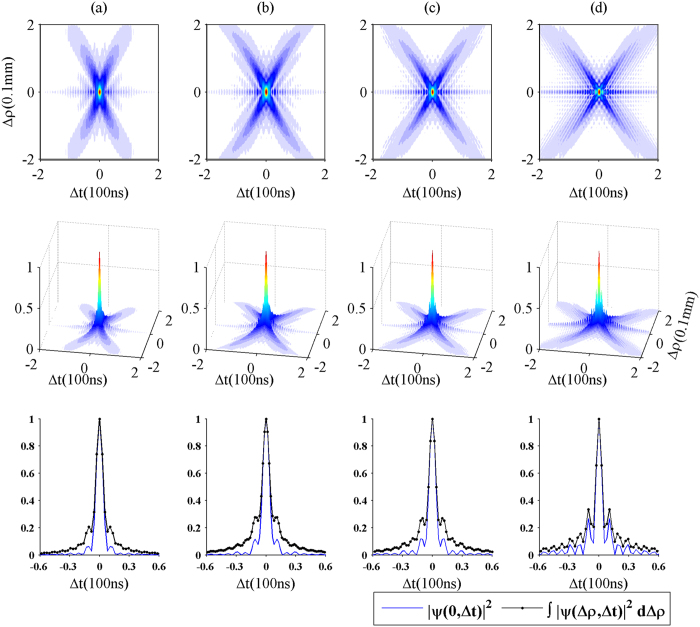
The first row shows the 2D cut of 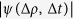 for different densities of the atomic medium: (**a**) 10^17^ atoms/m^3^, (**b**) 2 × 10^17^ atoms/m^3^, (**c**) 3 × 10^17^ atoms/m^3^, and (**d**) 4 × 10^17^ atoms/m^3^. All other parameters are the same as the ones in Fig. 2. The second row reports the corresponding 3D structures of the two-photon amplitude 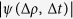. The last row compares the probability distribution when the paired photons are collected from the same position (blue solid line), with the integrated probability distribution obtained without discriminating their positions (black dotted line).

In Fig. 4, the upper panels plot the two-dimensional structures of the biphoton amplitudes for different densities of the atomic medium, and the central panels represent their corresponding three-dimensional structures of the biphoton amplitudes. The lower panels show the temporal profiles of the probability distribution 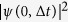 and the integrated probability distribution 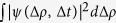 without discriminating the positions of paired photons.

As shown in Fig. (4), with the increase of the atomic density, the X envelope extends along the temporal axis, and meanwhile more oscillations arise around the X envelope. As discussed before, the X structure of the biphoton amplitude is under control of the FWM gain *g* and the phase mismatching 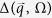. We note from (12) and (18) that the phase mismatching increases with increasing density *N* and the FWM gain 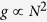 approximately. Therefore, the phase matching and the gain play a more and more important role in the biphoton correlation, with increasing atomic density. In consequence, the X envelope begins to expand, and the angle of the two wings of the X envelope changes (see Fig. 4). This can be understood as follows. From (20), we realize that 
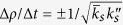
 and the quantity 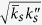 increases with increasing *N*. So, the angle between the two wings varies with increasing atomic density. Since the group velocities of the twin photons decrease with the increase of the atomic density, the photon pairs need to spend more time on propagating from the input plane to the output plane. Therefore, the delayed time between them becomes longer and longer, which makes the X envelope stretch out.

Moreover, the oscillations around the X structure become more obvious with increasing the atomic density, this can be explained as follows. With increasing atomic density, the interference effect between the two FWM processes becomes stronger, leading to the appearance of more oscillations, concentrated around the region for Δ*ρ* = 0, as shown in Fig. 4. However, if the density is extremely high, the slope Δ*ρ*/Δ*t* will be close to zero and the two wings of the X envelope will fold. In this situation, the X structure is not inseparable in the spatiotemporal domain anymore and the biphoton amplitude is mainly controlled by the FWM gain. Therefore, it is not difficult to find that the particular structure of biphoton amplitude is a consequence of the competition between the phase-matching condition and the FWM gain.

In addition, we plot the probability distribution 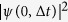 for Δ*ρ* = 0 and the integrated probability distribution 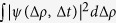 in the lower panels of Fig. (4). With increasing atomic density, the difference of the two standard deviations diminishes. This implies that pair photons tend to exit the output plane at the same position. So the biphoton correlation will be factorizable in space and time, if the density is large enough, as discussed before. Table 2 shows the standard deviations of the 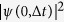 and the 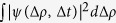. It can be seen that the effects of the atomic density on the two standard deviations is more obvious than the effects of the atomic length on them. The main reason is that, the FWM gain is gradually dominant for the profile of the biphoton amplitude with increasing the density, and more twin photons created and then exit the output plane in a time order. That makes the secondary oscillations enhanced in the lower panels of Fig. (4) and in turn results in the greater increase of the standard deviation of the two distributions compared with that in Table 1.

## Discussion and Conclusion

In summary, we have studied the spatiotemporal correlation of the biphoton created in a cold atomic medium with the FWM process. Under the conditions of the paraxial approximation and the quasi-monochromatic approximation, we first derive an expression for the biphoton probability amplitude, which is nonseparable in the space-time domain. Then we calculate the biphoton amplitude numerically, and find that the biphoton amplitude shows an X geometry structure. Such an X structure consists of two components, one is the X envelope caused by the particular phase matching, the other is the oscillations around the X envelope caused by the interference effect of the FWM processes. In fact, the complete X structure of the biphoton correlation is a consequence of the competition between the phase matching mechanism and the FWM gain mechanism. As shown in Fig. 3, when the length of the atomic medium is short, the X-shaped spatiotemporal structure is not clear. As the atomic medium becomes longer, the phase matching makes a great contribution to the spatiotemporal structure of the biphoton correlation, which leads to the obvious X envelope in Fig. 3(d). As shown in Fig. 4, when the atomic density and the FWM gain are not large, the properties of the biphoton correlation are mainly under control of the phase matching. However, with the increase of the atomic density, the FWM gain begins to play an important role in the biphoton correlation, instead of the phase matching, and meanwhile more and more oscillations around the X envelope appear, which blurs the X envelope of the biphoton correlation.

We have discussed the probability distribution in the case of Δ*ρ* = 0 and the integrated probability distribution, as well as their standard deviations. We find that when the medium is thin or the atomic density is large, the curves of the probability distribution for Δ*ρ* = 0 and the integrated probability distribution match well. The influences of the length and density of the medium on the standard deviation of the integrated probability distribution are always greater than that on the standard deviation of the probability distribution for Δ*ρ* = 0.

In conclusion, this work shows that by changing the length and density of the atomic medium, the spatiotemporal properties of the biphoton correlation can be modulated, which may be used in quantum precision measurement, quantum imaging, and quantum information processing.

## Additional Information

**How to cite this article:** Zhang, D. and Zhang, Z. The X-like shaped spatiotemporal structure of the biphoton entangled state in a cold two-level atomic ensemble. *Sci. Rep.* **7**, 42373; doi: 10.1038/srep42373 (2017).

**Publisher's note:** Springer Nature remains neutral with regard to jurisdictional claims in published maps and institutional affiliations.

## Supplementary Material

Supplementary Information

## Figures and Tables

**Table 1:** *σ*_1_ and *σ*_2_ are the standard deviations of the probability distribution 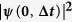 and the integrated probability distribution 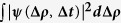 , respectively, for different lengths of the atomic medium.

	L = 2 mm	L = 5 mm	L = 10 mm	L = 20 mm
*σ*_1_	10.3 ns	10.3 ns	10.7 ns	11.9 ns
*σ*_2_	21.7 ns	30.7 ns	34.4 ns	35.9 ns

**Table 2:** *σ*_3_ and *σ*_4_ are the standard deviations of the the probability distribution 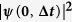 and the integrated probability distribution 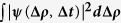 , respectively, for different densities of the atomic medium.

	N = 10^17^ m^−3^	N = 2 × 10^17^ m^−3^	N = 3 × 10^17^ m^−3^	N = 4 × 10^17^ m^−3^
*σ*_3_	9.4 ns	10.7 ns	12.9 ns	18.9 ns
*σ*_4_	26.9 ns	34.4 ns	41.5 ns	52.1 ns
