# Supplementary material for: The X-like shaped spatiotemporal structure of the biphoton entangled state in a cold two-level atomic ensemble
Source: Sci Rep. 2017 Feb 20;7:42373. doi: 10.1038/srep42373 (PMC5317000; doi:10.1038/srep42373)
Supplement: Supplementary Information [file srep42373-s1.pdf]

The X-like shaped spatiotemporal structure of the biphoton entangled state in a cold two-level atomic ensemble

Dasen Zhang, Zhiming Zhang

Supplementary Information

The derivation from (11) to (12) and (13)

By expanding (11), we can get

$$\dot{a}_j(z,\vec{\rho},t) = i\varpi_j a_j(z,\vec{\rho},t) + \frac{i}{\hbar}[H_0, a_j(z,\vec{\rho},t)] + \frac{i}{\hbar}[H_{int}, a_j(z,\vec{\rho},t)], (j = s, as). \tag{S1}$$

To evaluate the right-hand side of equation (S1) , it is necessary to know the commutation relation of the operators $a_j(z',\vec{\rho}',t)$ and $a_j^\dagger(z',\vec{\rho}',t)$ at the same time. Using equations (4) and (5), we can get

$$\left[a_j(z,\vec{\rho},t), a_j^\dagger(z',\vec{\rho}',t)\right] = c\widetilde{\delta}_j(\vec{r}-\vec{r}'), (j = s, as), \tag{S2}$$

where $\widetilde{\delta}_j(\vec{r}-\vec{r}') = \int \frac{dk_{jz}}{2\pi} \int \frac{d\vec{q}_j}{(2\pi)^2} \frac{\omega(k_j)}{\bar{k}_j} e^{i\left(k_{jz}-\bar{k}_j\right)(z-z')+i\vec{q}_j\cdot(\vec{\rho}-\vec{\rho}')}$. Using the commutation relation (S2), equation (S1) can be written as

$$\dot{a}_j(z,\vec{\rho},t) = i\varpi_j a_j(z,\vec{\rho},t) - i\varpi_j \int_V d\vec{r}'\widetilde{\delta}_j(\vec{r}-\vec{r}')a_j(z',\vec{\rho}',t) - \widetilde{g}(\Omega)\int_V d\vec{r}' e^{i\Delta z}\widetilde{\delta}_j(\vec{r}-\vec{r}')a_l^\dagger(z',\vec{\rho}',t), (j,l = s,as \text{ and } j \neq l), \tag{S3}$$

where $\Delta = 2k_p - k_{asz} - k_{sz}$ and $\widetilde{g}(\Omega) = \left(\frac{i\sqrt{\varpi_s\varpi_{as}}}{2}\right) E_p^2 \chi^{(3)}(\Omega)$.

To simplify equation (S3), we perform the Fourier transform:

$$a_j(\kappa,\vec{q},\Omega) = \int dz e^{-i\kappa z} \int d\vec{\rho} e^{-i\vec{q}\cdot\vec{\rho}} \int dt e^{i\Omega t} a_j(z,\vec{\rho},t), \quad (j = s, as). \tag{S4}$$

By substituting equation (S4) into equation (S3) where we need to use the identities $\delta\left(\kappa - k_{jz} + \bar{k}_j\right) = \int dz' e^{i\left(\kappa - k_{jz} + \bar{k}_j\right)z'}$ and $\delta(\vec{q}-\vec{q}_j) = \int d\vec{\rho}' e^{i(\vec{q}-\vec{q}_j)\cdot\vec{\rho}'}$, we can get

$$-i\Omega a_j(\kappa,\vec{q},\Omega) = -i\left(\omega(k_j) - \varpi_j\right) a_j(\kappa,\vec{q},\Omega) - \widetilde{g}(\Omega) a_l^\dagger(-\kappa+\Delta,-\vec{q},-\Omega), (j,l = s,as \text{ and } j \neq l), \tag{S5}$$

here $\omega(k_j)$ corresponds to the Stokes or anti-Stokes wave vector $\vec{k}_j$=$(\vec{q}, k_{jz}+\kappa)$. In above equation, we are only interested in the process of parametric interaction, so we try to deal with the part of the parametric interaction from equation (S5) by introducing the new field amplitudes as

$$a_j(z,\vec{q},\Omega) = \varepsilon_j(z,\vec{q},\Omega) e^{i\left(k_{jz}(\vec{q},\Omega) - \bar{k}_j\right)z}, \tag{S6}$$

$$a_j^\dagger(z,\vec{q},\Omega) = \varepsilon_j^\dagger(z,\vec{q},\Omega) e^{-i\left(k_{jz}(\vec{q},\Omega) - \bar{k}_j\right)z}, \tag{S7}$$

where

$$k_{jz}(\vec{q},\Omega) = \sqrt{\left|\vec{k}_j(\varpi_j+\Omega)\right|^2 - |\vec{q}|^2}, \tag{S8}$$

$$\varepsilon_j(\kappa,\vec{q},\Omega) = a_j\left(\kappa + k_{jz}(\vec{q},\Omega) - \bar{k}_j, \vec{q}, \Omega\right), \tag{S9}$$

$$\varepsilon_j^\dagger(-\kappa,-\vec{q},-\Omega) = a_j^\dagger\left(-\kappa + k_{jz}(-\vec{q},-\Omega) - \bar{k}_j, -\vec{q}, -\Omega\right). \tag{S10}$$

Using the new operators $\varepsilon_j(\kappa,\vec{q},\Omega)$, we can rewrite equation (S5) as

$$-i\Omega\varepsilon_j(\kappa,\vec{q},\Omega) = -i\left(\omega\left(k_j'\right) - \varpi_j\right)\varepsilon_j(\kappa,\vec{q},\Omega) - \widetilde{g}(\Omega)\varepsilon_l^\dagger(-\kappa - \Delta(\vec{q},\Omega), -\vec{q}, -\Omega), (j,l = s,as \text{ and } j \neq l), \tag{S11}$$

where $\vec{k}'_j = (\kappa + k_{jz}(\vec{q},\Omega), \vec{q})$ and the phase-mismatch function $\Delta(\vec{q},\Omega) = k_{asz}(-\vec{q},-\Omega) + k_{sz}(\vec{q},\Omega) - 2k_p$. With the paraxial and quasimonochromatic approximations, we arrive at

$$\omega\left(k'_j\right) = \overline{\omega}_j + \Omega + V_j\kappa, \tag{S12}$$

where $V_j = \frac{\partial\omega(k)}{\partial k}|_{k=\bar{k}_j}$ is the group velocity of the Stokes or anti-Stokes wave in the cold atomic medium. Considering the relation $\bar{k}_s = \bar{k}_{as}$, we take $V_g = V_s = V_{as}$ in the calculation below. By substituting equation (S12) into equation (S11), we can obtain

$$i\kappa\varepsilon_j(\kappa,\vec{q},\Omega) = g(\Omega)\,\varepsilon_l^\dagger(-\kappa - \Delta(\vec{q},\Omega), -\vec{q}, -\Omega), \quad (j,l = s, as \text{ and } j \neq l), \tag{S13}$$

where $g(\Omega) = -\frac{\tilde{g}(\Omega)}{V_g}$. With the help of the relation $\varepsilon_j(\kappa,\overrightarrow{q},\Omega) = \int dz e^{-i\kappa z}\varepsilon_j(z,\overrightarrow{q},\Omega)$, equation (S13) can be finally simplified to (12) and (13).

The input-output solutions of (12) and (13)

The input-output relations of (12) and (13) are given by:

$$\overline{\varepsilon}_s(L,\vec{q},\Omega) = U_s(\vec{q},\Omega)\,\overline{\varepsilon}_s(0,\vec{q},\Omega) + V_s(\vec{q},\Omega)\,\overline{\varepsilon}_{as}^\dagger(0,-\vec{q},-\Omega), \tag{S14}$$

$$\overline{\varepsilon}_{as}(L,-\vec{q},-\Omega) = U_{as}(-\vec{q},-\Omega)\,\overline{\varepsilon}_{as}(0,-\vec{q},-\Omega) + V_{as}(-\vec{q},-\Omega)\,\overline{\varepsilon}_s^\dagger(0,\vec{q},\Omega), \tag{S15}$$

where

$$\overline{\varepsilon}_j(L,\vec{q},\Omega) = e^{ik_{jz}(\vec{q},\Omega)L}\varepsilon_j(L,\vec{q},\Omega), \qquad (j = s, as), \tag{S16}$$

$$U_s(\vec{q},\Omega) = e^{i\Delta_s(\vec{q},\Omega)L/2}\left\{\cosh[\Gamma(\vec{q},\Omega)L] - \frac{i\Delta(\vec{q},\Omega)}{2\Gamma(\vec{q},\Omega)}\sinh[\Gamma(\vec{q},\Omega)L]\right\}, \tag{S17}$$

$$V_s(\vec{q},\Omega) = e^{i\Delta_s(\vec{q},\Omega)L/2}\frac{g(\Omega)}{\Gamma(\vec{q},\Omega)}\sinh[\Gamma(\vec{q},\Omega)L], \tag{S18}$$

$$U_{as}(-\vec{q},-\Omega) = e^{i\Delta_{as}(\vec{q},\Omega)L/2}\left\{\cosh[\Gamma(\vec{q},\Omega)L] - \frac{i\Delta(\vec{q},\Omega)}{2\Gamma(\vec{q},\Omega)}\sinh[\Gamma(\vec{q},\Omega)L]\right\}, \tag{S19}$$

$$V_{as}(-\vec{q},-\Omega) = e^{i\Delta_{as}(\vec{q},\Omega)L/2}\frac{g(\Omega)}{\Gamma(\vec{q},\Omega)}\sinh[\Gamma(\vec{q},\Omega)L], \tag{S20}$$

$$\Delta_s(\vec{q},\Omega) = k_{sz}(\vec{q},\Omega) - k_{asz}(-\vec{q},-\Omega) + 2k_p, \tag{S21}$$

$$\Delta_{as}(\vec{q},\Omega) = -k_{sz}(\vec{q},\Omega) + k_{asz}(-\vec{q},-\Omega) + 2k_p, \tag{S22}$$

$$\Gamma(\vec{q},\Omega) = \sqrt{|g(\Omega)|^2 - \frac{1}{4}(\Delta(\vec{q},\Omega))^2}, \tag{S23}$$

where $\overline{\varepsilon}_s(L,\vec{q},\Omega)$ and $\overline{\varepsilon}_{as}(L,\vec{q},\Omega)$ are the amplitude operators on the output face and the phase mismatch function is $\Delta(\vec{q},\Omega) = 2k_p - k_{asz}(-\vec{q},-\Omega) - k_{sz}(\vec{q},\Omega)$. In addition, it is necessary to keep the commutation relations

$$\left[\overline{\varepsilon}_j(0,\vec{q},\Omega), \overline{\varepsilon}_l^\dagger(0,\vec{q}',\Omega')\right] = \delta_{jl}\delta(\vec{q}-\vec{q}')\delta(\Omega-\Omega'), (j,l = s, as), \tag{S24}$$

from the input plane to the output plane, so equations (S17∼S20) need to satisfy the following forms:

$$\left|U_j(\vec{q},\Omega)\right|^2 - \left|V_j(\vec{q},\Omega)\right|^2 = 1, \tag{S25}$$

$$U_j(\vec{q},\Omega)V_l(-\vec{q},-\Omega) = U_l(-\vec{q},-\Omega)V_j(\vec{q},\Omega), (j,l = s, as \text{ and } j \neq l). \tag{S26}$$

The derivation from (14) to (15)

By means of the Fourier transform of $\overline{\varepsilon}_j(L,\vec{\rho},t)$, we can get

$$\psi(\vec{\rho},t,\vec{\rho}',t') = \int \frac{d^2\vec{q}d\Omega}{(2\pi)^3} \int \frac{d^2\vec{q}'d\Omega'}{(2\pi)^3} \delta(\vec{q}+\vec{q}')\,\delta(\Omega+\Omega')\left\langle \overline{\varepsilon}_s(L,\vec{q},\Omega)\overline{\varepsilon}_{as}(L,\vec{q}',\Omega')\right\rangle e^{i(\vec{q}\cdot\vec{\rho}-\Omega t)}e^{i(\vec{q}'\cdot\vec{\rho}'-\Omega' t')}, \tag{S27}$$

where the δ functions come from the energy conservation and the momentum conservation. So we can obtain

$$\psi(\vec{\rho},t,\vec{\rho}',t') = \int \frac{d^2\vec{q}d\Omega}{(2\pi)^3} e^{i[\vec{q}\cdot(\vec{\rho}-\vec{\rho}')-\Omega(t-t')]}\langle \overline{\varepsilon}_s(L,\vec{q},\Omega)\overline{\varepsilon}_{as}(L,-\vec{q},-\Omega)\rangle\,. \tag{S28}$$

Considering that the above equation include the output operators $\overline{\varepsilon}_s(L,\vec{q},\Omega)$ and $\overline{\varepsilon}_{as}(L,-\vec{q},-\Omega)$, we can apply the input-output relations to simplify ψ:

$$\psi(\vec{\rho}-\vec{\rho}',t-t') = \int \frac{d^2\vec{q}d\Omega}{(2\pi)^3} \left\langle \overline{\varepsilon}_s(0,\vec{q},\Omega)\,\overline{\varepsilon}^{\dagger}_{as}(0,\vec{q},\Omega)\right\rangle U_s(\vec{q},\Omega)\,V_{as}(-\vec{q},-\Omega)\,e^{i[\vec{q}\cdot(\vec{\rho}-\vec{\rho}')-\Omega(t-t')]}. \tag{S29}$$

So we obtain

$$\psi(\vec{\rho}-\vec{\rho}',t-t') = \int \frac{d^2\vec{q}d\Omega}{(2\pi)^3} U_s(\vec{q},\Omega)V_{as}(-\vec{q},-\Omega)e^{i[\vec{q}\cdot(\vec{\rho}-\vec{\rho}')-\Omega(t-t')]}. \tag{S30}$$

By substituting equations (S17) and (S20) into equation (S30), we finally obtain (15).
